# A Case Report of Aggressive Chronic Rhinosinusitis with Nasal Polyps Mimicking Sinonasal Malignancy

**DOI:** 10.1155/2019/3725720

**Published:** 2019-06-09

**Authors:** S. Velegrakis, N. Chatzakis, E. Prokopakis, M. Papadakis, E. Panagiotaki, M. Doulaptsi, A. Karatzanis

**Affiliations:** Department of Otorhinolaryngology, University of Crete School of Medicine, Heraklion, Crete, Greece

## Abstract

**Introduction:**

Cases of extensive nasal polyps rarely occur and may mimic more aggressive lesions of the nose and paranasal sinuses. A case of extensive nasal polyposis with unusually aggressive behavior and its management is presented.

**Presentation of Case:**

A 27-year-old male patient visited the emergency department of a tertiary center, complaining of recurrent episodes of epistaxis. The patient presented with a large polypoid lesion protruding from the right nostril and producing asymmetry of the face. Diagnostic imaging illustrated a lesion of the right maxillary sinus producing excessive bone remodeling and extension into neighboring structures in every direction. Fine limits were noted, however, with no invasive characteristics. Biopsy under local anesthesia was performed, showing findings consistent with nonspecific inflammation. Open surgery through a lateral rhinotomy under general anesthesia was performed, and the mass was readily mobilized and removed. No macroscopic invasion of neighboring structures was noted. Permanent histology confirmed the diagnosis of nasal polyposis. Postoperative follow-up has shown no evidence of recurrence after 12 months.

**Conclusion:**

Nasal polyps do not typically expand in an aggressive manner, producing bone resorption or extending into neighboring structures. However, nasal polyposis should be included in the differential diagnosis of nasal tumors with such behavior.

## 1. Introduction

Sinonasal tumors are rare entities with distinctive clinical, etiological, and pathological features. Nasal and paranasal cavities, although small spaces, represent complex areas, where a wide range of benign and malignant tumors may occur. Primary benign and malignant tumors account for approximately 3% of all head and neck neoplasms [1–3].The diagnosis and treatment of these tumors is challenging because of their low incidence, histological diversity, and indolent clinical course. Malignancies have a variable prognosis depending on histology, origin, and clinical stage. Proximity to vital anatomical structures makes their management quite complex [2]. High morbidity and mortality are generally expected.

Nasal polyposis develops in 0.2-1% of the general population and concerns all races, increasing with age [3]. Prevalence is much higher in individuals with comorbidities such as asthma, aspirin intolerance, or cystic fibrosis [4]. In some rare cases, nasal polyposis may behave aggressively and mimic other pathologies of nasal-paranasal cavities [5]. Pathogenesis of sinonasal polyposis remains unclear, but it has been shown that eosinophil-dominated inflammation plays a major role in the development and progression of the disease.

In this case report, we present a patient with aggressive nasal polyposis causing bone remodeling and extension into neighboring structures, and thus mimicking much more aggressive disease. We present in detail the clinical evaluation as well as surgical technique and postoperative follow-up. This case report is compliant with the SCARE Guidelines and PROCESS Guidelines.

## 2. Case Presentation

A 27-year-old male, with Crouzon syndrome phenotype, visited the emergency department of a tertiary referral center, reporting multiple episodes of epistaxis in the past few days. The patient also reported nasal obstruction and impaired nasal breathing for the previous several months. Rest of the medical history was free. On clinical examination, a polypoid lesion protruding from the right nostril was noted. In addition, asymmetry of the face and projection of the ipsilateral canine fossa were evident.

**Figure 1:**
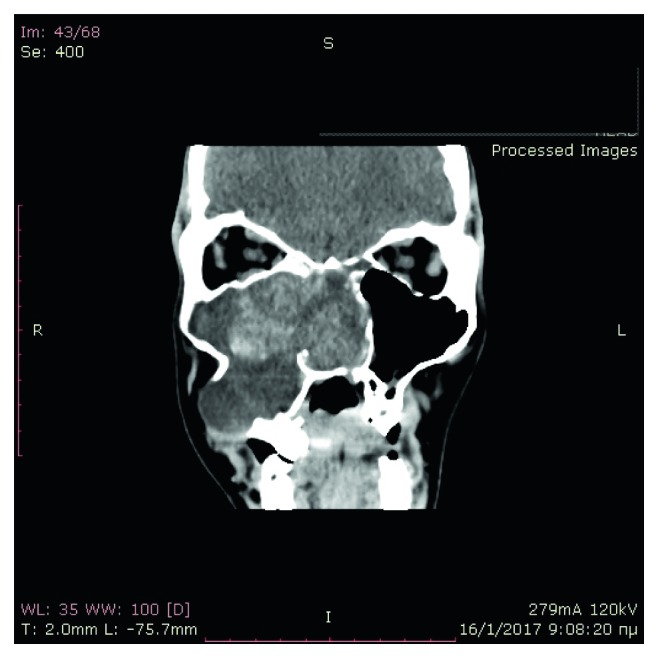
Preoperative CT, coronal plane.

**Figure 2:**
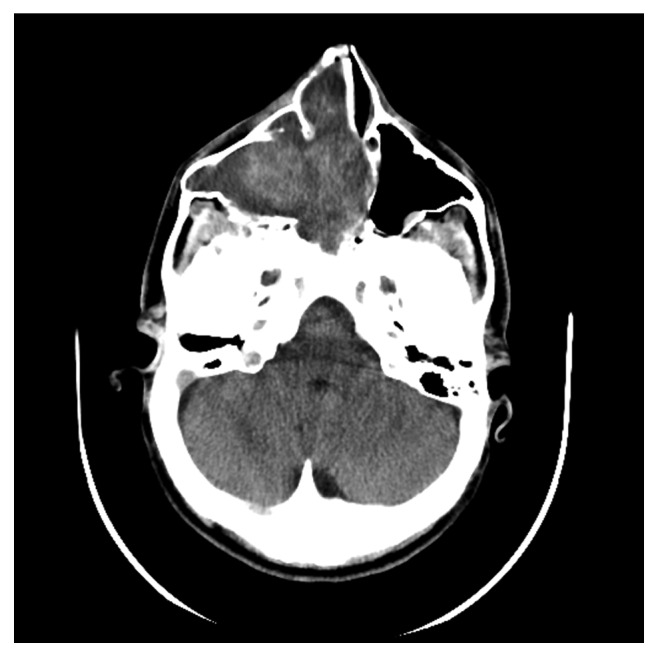
Preoperative CT, axial plane.

**Figure 3:**
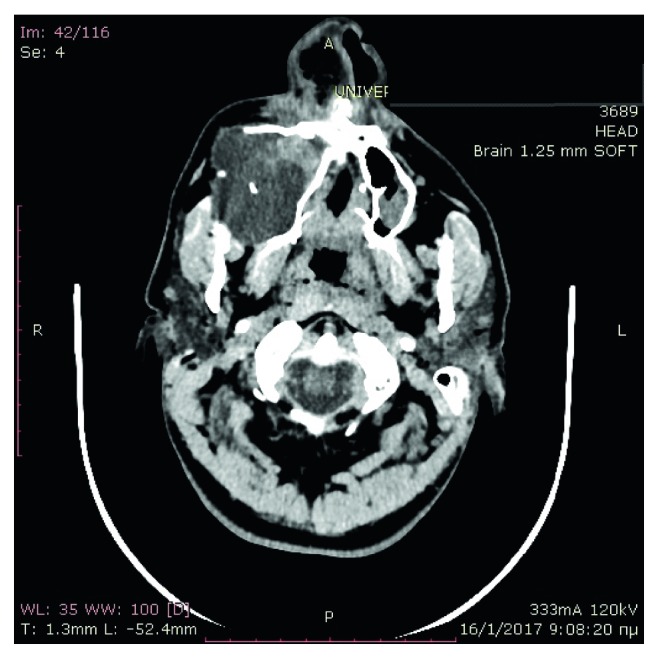
Preoperative CT, axial plane.

**Figure 4:**
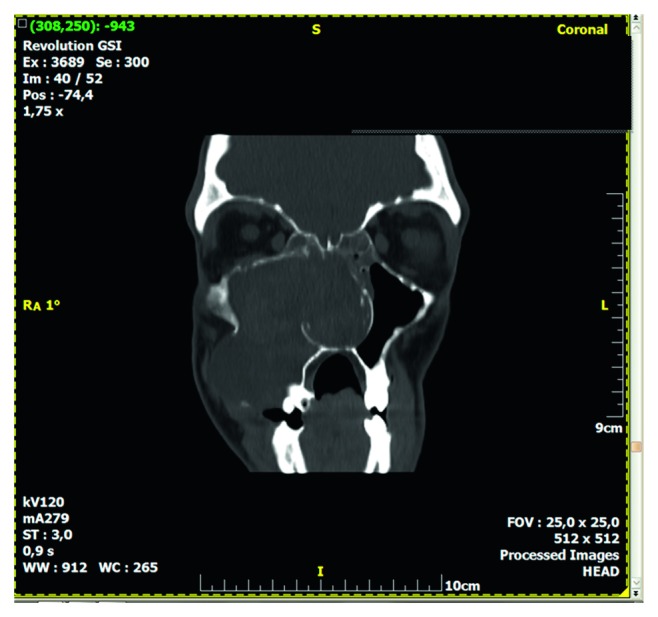
Preoperative CT, coronal plane, bone window.

**Figure 5:**
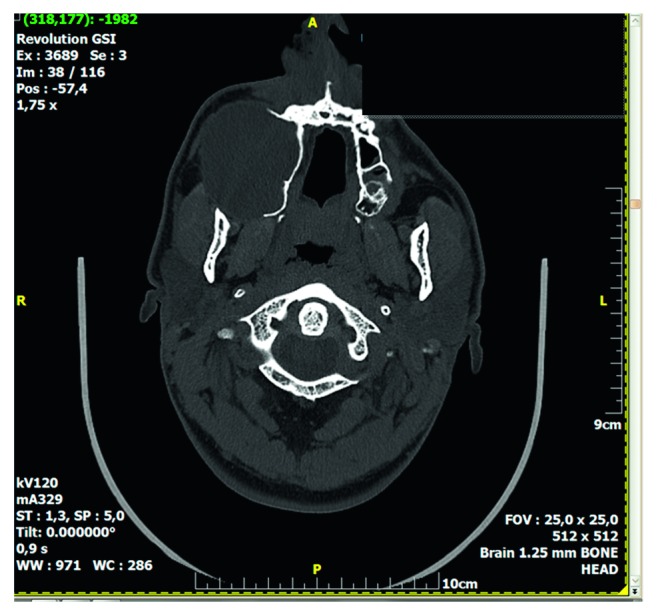
Preoperative CT, axial plane, bone window.

Computed tomography of the paranasal sinuses showed an inhomogeneous soft-tissue mass, which completely occupied the right nasal cavity, maxillary sinus, and anterior and posterior ethmoidal cells. The lesion produced extensive bone remodeling of the right maxillary sinus with complete absence of its anterior wall, as well as erosion of the posterior wall and entry of the lesion in the pterygopalatine fossa. There was also erosion of the ipsilateral lower as well as median orbital wall, and entry of the lesion in the orbital cavity. Despite its large size, the lesion seemed to be well defined without invasive characteristics (Figures 1–5).

**Figure 6:**
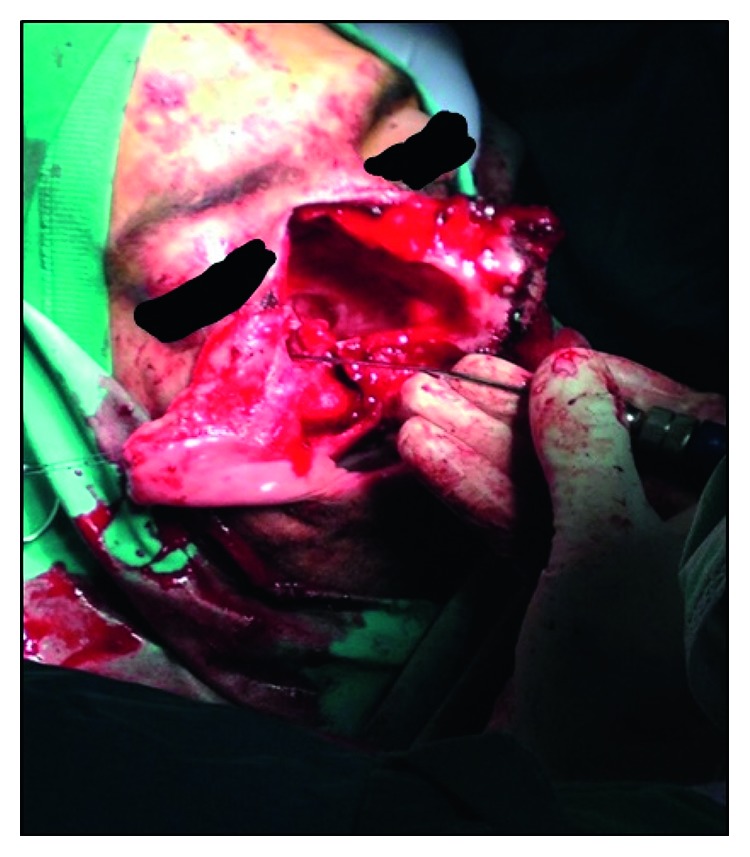
Intraoperative overview.

**Figure 7:**
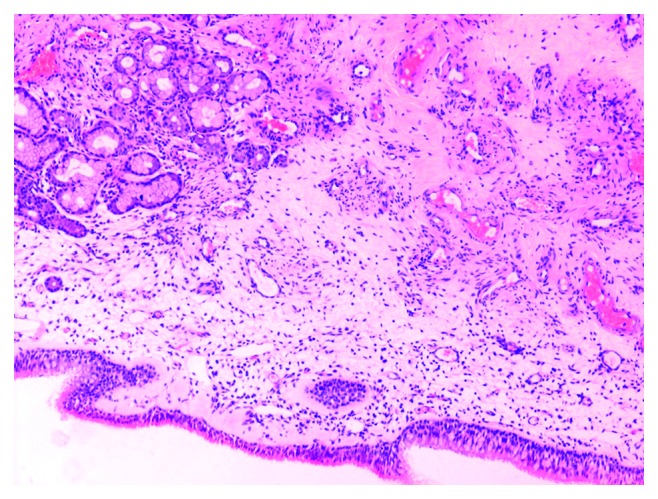
Final histological examination.

**Figure 8:**
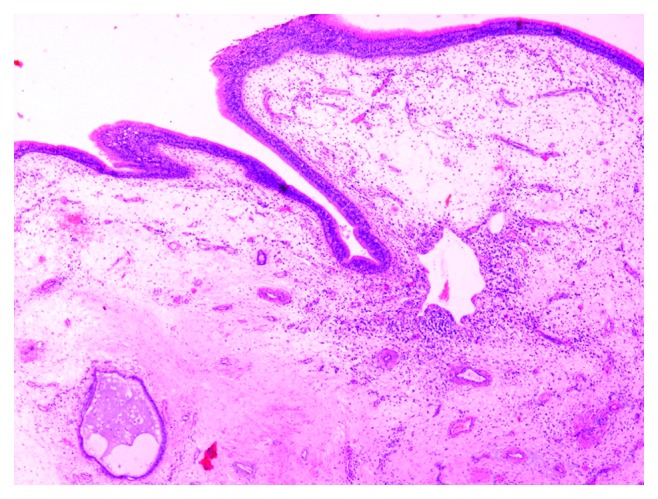
Final histological examination.

**Figure 9:**
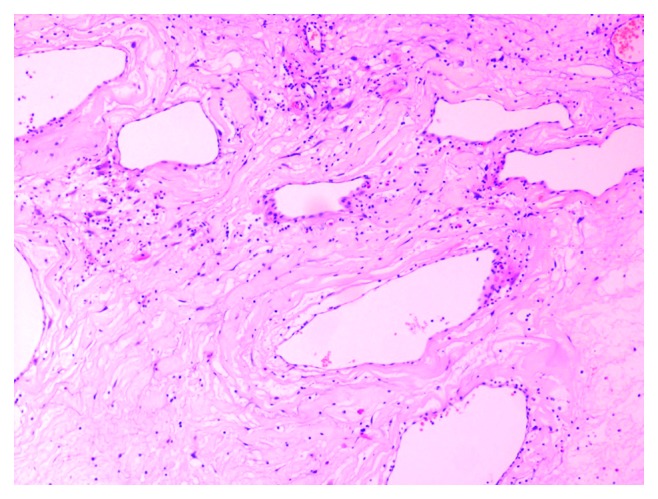
Final histological examination.

**Figure 10:**
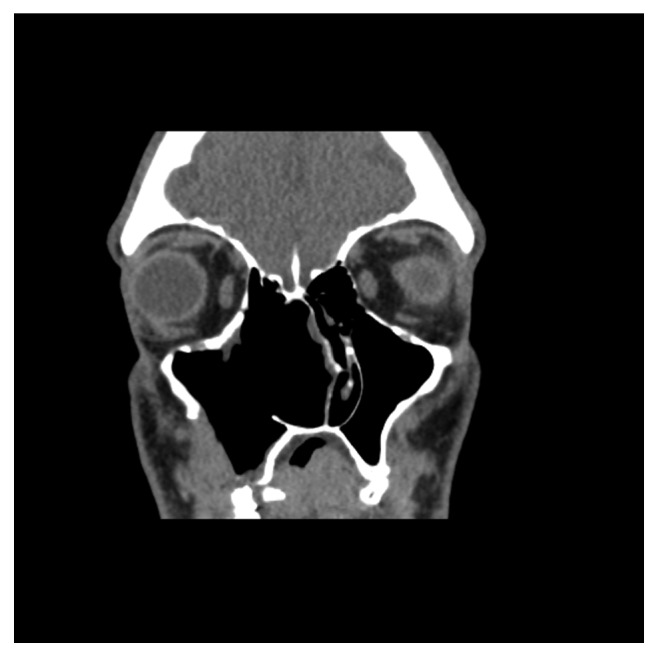
Postoperative CT, coronal plane.

**Figure 11:**
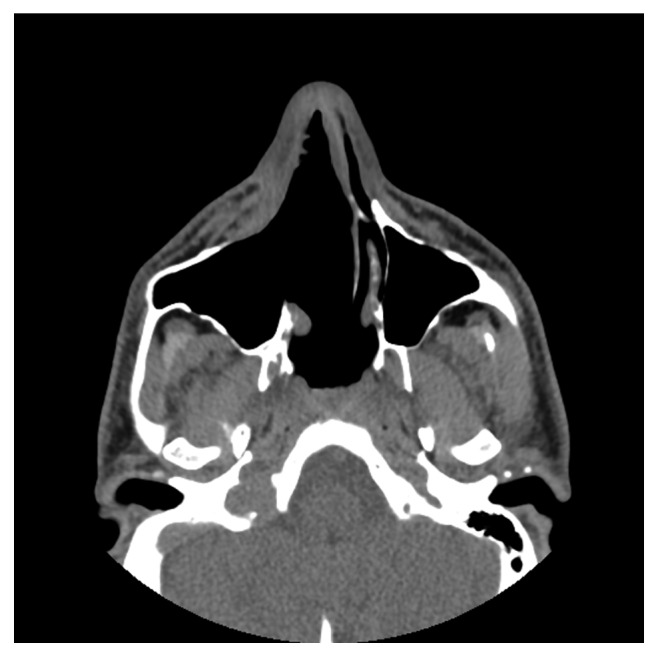
Postoperative CT, axial plane.

Routine laboratory tests were within normal range. Preoperative maxillofacial consultation excluded pathology of odontogenic origin. The patient underwent a biopsy under local anesthesia, and the findings showed nonspecific inflammation. Open surgery under general anesthesia was undertaken via lateral rhinotomy and medial maxillectomy (Figure 6). The maxillary sinus mucosa was completely replaced by inflammatory tissue simulating a benign mass. This mass was readily mobilized and dissected free from surrounding tissues within the orbit and pterygopalatine fossa, as no macroscopic invasion of any neighboring structures was noted. Histopathological examination revealed typical nasal polyposis with mixed population of eosinophils, neutrophils, and macrophages, with no evidence of fungal invasion (Figures 7–9). Antibiotic and corticosteroid treatment was performed for a short period postoperatively. Local nasal mometasone furoate was used for 2 months after surgery. Intensive saline solution irrigations were additionally administered. There are no clinical/radiological signs or symptoms of recurrence 12 months postoperatively (Figures 10 and 11).

## 3. Discussion

Nasal polyposis is a very common entity with prevalence between 6 and 11% in the Western world. Cases with aggressive behavior [6, 7], however, are rare [8, 9]. In fact, only a few cases with bony destruction and erosion have been reported. Turel et al. reported a case of nasal polyposis resulting in fibro-osseous thickening of sinonasal, maxillofacial bones, and proptosis [9]. Arvind et al. presented a case of osteolytic nasal polyps of the maxillary sinus, mimicking malignancy with invasion to the facial soft tissue [10]. Majitha et al. presented intracranial expansion of nasal polyps in patients with Samter's triad [2]. Rejowski et al. reported a case of nasal polyposis with bony destruction and acute bilateral visual loss due to optic nerve compression [11]. Midline lesions, such as Wegener's granulomatosis and T-cell lymphoma, may also cause extensive bone erosion and soft-tissue involvement and should always be considered in the differential diagnosis. These clinical entities typically first involve the nasal septum, show pathognomonic features in immunocytochemistry, and tend to reoccur without additional treatment [12].

Sinonasal angiomatous polyp is a rare variant of sinonasal polyp that may mimic inverted papilloma, juvenile angiofibroma, and malignant tumors in its clinical and radiological aspects [13]. The CT and MR imaging typically show expansile sinonasal-occupying lesions with bony destruction and obstructive sinusitis in adjacent sinus cavities. Histologically, this pathology is characterized by extensive vascular proliferation and angiectasis, resulting in venous stasis, thrombosis, and infarction [14]. Despite aggressive clinical characteristics, most cases of sinonasal angiomatous polyps may be treated with conservative surgical excision and recurrences are rare. Although clinical and radiological features of our case could have been attributed to a sinonasal angiomatous polyp, such a diagnosis was not confirmed by permanent histology.

In addition to nasal polyps, other inflammatory conditions of the nose may occasionally follow an aggressive clinical course. Vorasubin et al. reported a rare case of invasive actinomycosis presenting with extensive midface destruction involving the maxilla and paranasal sinuses, with mucosal necrosis mimicking an aggressive neoplasm. Although this is very rare condition, it should be included in the differential diagnosis [15].

Clinical evaluation of a patient presenting with a nasal mass may be quite complex. Endoscopy, imaging studies, and evaluation of the symptoms are crucial in differential diagnosis and therapeutic planning. Computed tomography (CT) is the gold standard in the radiologic investigation of the paranasal sinuses for diagnosis of sinonasal lesions as well as pre- and postsurgical assessment [16]. Computed tomography may, among other things, reliably show sinonasal bone expansion, erosion, and thickening. There seems to be relevance between imaging studies and disease severity [17]. Optimal imaging is needed to determine the origin and the distribution pattern of a tumor. If there is a suspicion regarding the development of orbital and intracranial invasion or complications, magnetic resonance imaging (MRI) has higher sensitivity and specificity than CT scanning. Even with the combination of the most modern imaging modalities, paranasal masses may be difficult to diagnose due to overlap among radiological features [18]. Definitive diagnosis, with few exceptions, requires biopsy and should be established from histopathological examination [5].

The most frequent reported symptoms of nasal and paranasal masses at the time of diagnosis are nasal congestion, headache, nasal discharge, diplopia, facial swelling, proptosis, auditory impairment, and epistaxis [19]. Nasal congestion, headache, nasal discharge, and epistaxis are common between nasal benign and malignant pathologies. Patients may present with nonspecific symptoms of sinusitis, nasal bleeding, or other symptoms even in cases where the lesion reaches the skullbase or the orbit [18].

Surgical treatment protocols have evolved from extensive craniofacial resection to more conservative endoscopic sinus surgery in order to lower morbidity rates and improve treatment outcomes. With the development of functional endoscopic sinus surgery (FESS), indications for classical surgical procedures have been limited significantly. While the majority of patients may be adequately managed endoscopically, we highlight the importance of having the option of combined and open craniofacial approaches for extensive and complicated disease. Reported relapses after endoscopic surgery reach 60% for chronic sinusitis with nasal polyps, and some patients with frequent recurrences may benefit from classical approaches in order to achieve better disease control over prolonged periods of time [20]. Extent of the lesion in our case, history of repeated nose bleeding, and suspicion of more aggressive disease despite first biopsy results were the main reasons that led to the decision of an open surgical approach [21]. Judging from the final clinical outcome, however, with the knowledge of permanent histopathology, a less radical, combined surgical approach may have been considered as more appropriate to remove this lesion.

## 4. Conclusion

There is an overlap between symptoms, clinical signs, and imaging findings in many pathological entities of the nasal-paranasal cavities. Nasal polyps typically do not expand aggressively, leading to bone resorption and extension into neighboring spaces. However, nasal polyposis should always be included in the differential diagnosis of nasal tumors with such behavior. Extensive surgery may be warranted in these cases, and excellent results should be expected.

## Additional Points

We present a case report of nasal polyposis with unusually aggressive behavior. Nasal polyps do not frequently present with bone deformities and invasion into neighboring structures. There is an overlap in the appearance of nasal-paranasal cavity pathologies, and diagnostic differentiation is sometimes challenging.
